# Meta-analysis of colistin for the treatment of *Acinetobacter baumannii* infection

**DOI:** 10.1038/srep17091

**Published:** 2015-11-24

**Authors:** Zhijin Chen, Yu Chen, Yaogao Fang, Xiaotian Wang, Yanqing Chen, Qingsong Qi, Fang Huang, Xungang Xiao

**Affiliations:** 1 Department of Hospital Infection-Control, Affiliated Houjie Hospital, Guangdong Medical College, Dongguan, Guangdong 523945, China; 2 Department of Joint Surgery, Chenzhou NO.1 People’s Hospital, Hunan Province, 423000 P.R. China; 3 Department of Urology, Affiliated Hospital of Huzhou Teachers’ College, The First People’s Hospital of Hu zhou, Hu zhou, 313000, P.R. China

## Abstract

Multidrug resistant among *Acinetobacter baumannii* infection is associated with a high mortality rate and limits the therapeutic options. The aim of this study was to assess the safety and efficacy of colistin monotherapy vs. other single antibiotic therapy AND colistin-based combination therapy (with other antibiotics) vs. colistin alone for the treatment of *Acinetobacter baumannii* infection. Online electronic database were searched for studies evaluating colistin with or without other antibiotics in treatment of patients with drug-resistant *Acinetobacter baumannii* infection. Totally, twelve studies met the inclusion criteria. For colistin-based combination therapy, six articles including 668 patients were included. Our results showed that the overall clinical response did not differ significantly between colistin-based combination therapy and monotherapy (OR = 1.37, 95% CI = 0.86–2.19, P = 0.18). This insignificance was also detected in ICU mortality, length of stay and nephrotoxicity (P > 0.05). However, the colistin-based combination therapy was shown increasing the microbiological response (OR = 2.14, 95% CI = 1.48–3.07, P < 0.0001). For colistin monotherapy, six studies involving 491 patients were analyzed. The results were in concordance with the findings of the colistin-based combination therapy group. Our results suggest that colistin may be a promising therapy as safe and efficacious as standard antibiotics for the treatment of drug-resistant *Acinetobacter baumannii* infection.

*Acinetobacter baumannii* is an aerobic Gram-negative pathogen, which is often associated with nosocomial infections in the immunocompromised patient population^1^. In 1911, Beijerinck was the first to isolate and describe the organism that would now be recognized as Acinetobacter^2^. Most clinically significant isolates belong to the species *A. baumannii* or its close relatives, which together account for the vast majority of infections and hospital outbreaks involving Acinetobacter spp^3^. Recently, multidrug resistant *A. baumannii* (MDR-AB), which is resistant to all standard antimicrobial agents, began to expand, resulting in high treatment failure in some areas^4^. The presence of MDR-AB increases the prevalence of *A. baumannii* infection, and makes the choice of appropriate antimicrobial treatment difficult.

Colistin and tigecycline remain the only active antibiotics and have become the last resort of treatment for drug-resistant *A. baumannii* infection^5^. Previous meta-analysis conducted by Tasina *et al.* showed that tigecycline was not better than the usually used antimicrobial agents^6^, which have led to increased reliance on colistin in treating widespread drug-resistant *A. baumannii* infection. Colistin, a natural substance produced by Bacillus polymyxa subspecies colistinus, is a cationic lipopeptide^7^. It is rapidly bactericidal against Gram-negative bacteria by interacting with the lipid A moiety of lipopolysaccharide (LPS) to cause rupture of the outer membrane, and leading to cell permeability changes, leakage of the cellular content, and cell death^7^,^8^. Colistin sulfate and colistimethate sodium are the two commercially available forms. Recently, colistin has increasingly been used as salvage therapy, either alone or in combination with other antibiotics, for the treatment of severe infections in critically ill patients^9^. And it has been recommended in American Thoracic Society Guidelines as a therapeutic option for the treatment of VAP (Ventilator-associated pneumonia) caused by drug-resistant gram-negative organisms^10^. Recent study have also demonstrated that the use of higher doses of colistin, or colistin-based combination therapy (with other antibiotics), may prevent emerging resistance and preserve the activity of polymyxins against A. baumannii.

Though colistin is used in clinical practice, and is considered to be sub-optimal to beta-lactams, some reports have showed that it is very nephrotoxic and resistance to colistin has been reported in a particular area, from which the highest resistance rate was reported in Asia, followed by Europe^11^. Moreover, colistin heteroresistance and colistin resistance have been described in *A. baumannii*. Thus, we conducted this meta-analysis to systematically evaluate the safety and efficacy of colistin for the treatment of *A. baumannii* infection.

## Results

### Characteristics of studies included

**Figure 1:**
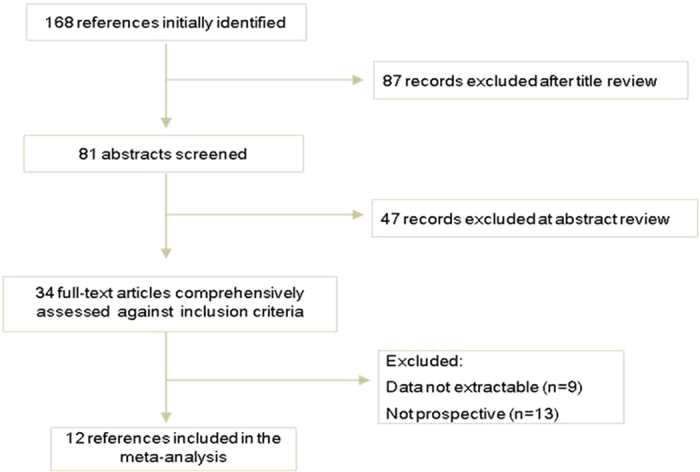
Flow chart demonstrating studies that were processed for inclusion in the447 meta-analysis.

As shown in Fig. 1, out of a total of initially reviewed 168 studies, only 12 studies including 1159 patients were considered eligible for inclusion in this meta-analysis. For colistin monotherapy, 6 studies involving 491 patients, were included. Of them, one was from Thailand^12^, one from Greece^13^, one from South Africa^14^, one from Spain^15^, one from Korea^16^ and one from China Taiwan^17^. For colistin-based combination therapy, 6 articles including 668 patients were contained. Four were from Turkey^18^,^19^,^20^,^21^, one from Korea^22^ and one from Italy^23^. Characteristics of the studies included in this analysis were presented in Table 1.

### Meta-analysis of colistin monotherapy

#### Clinical outcome

**Figure 2:**
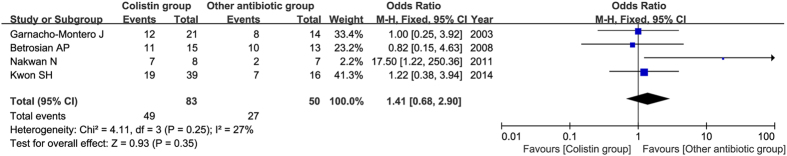
Clinical response of colistin monotherapy compared with control antibiotics.

Four studies reported the clinical response, including 133 patients (83 with colistin treatment, and 50 with other antibiotics). No statistical heterogeneity was observed among studies (P = 0.25, I^2^ = 27%), and the fixed-effects model was employed. Overall, our result found that the effective rate of clinical response in patients with colistin group (experimental group) was a little higher than that in other antibiotic group (control group) (59% versus 54%). However, the overall clinical response did not differ significantly between colistin group and control groups (OR = 1.41, 95% CI = 0.68–2.90, P = 0.35) as shown in Fig. 2.

### Microbiological outcome

**Figure 3:**
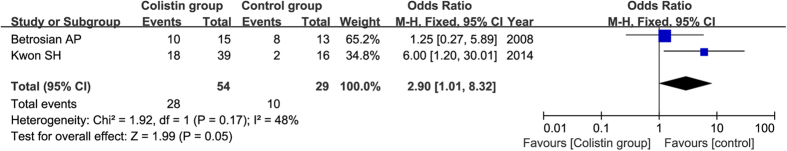
Forest plot for microbiological response between colistin group and control troup.

Two studies compared colistin with other antibiotics in terms of microbiological response, including 83 patients (54 with colistin treatment, 29 with other antibiotics). The result from these two studies showed that there was a significant difference between colistin group and control groups (OR = 2.90, 95% CI = 1.01–8.32, P = 0.05) as shown in Fig. 3. No statistical heterogeneity was found among studies (P = 0.17, I^2^ = 48%).

### Mortality

**Figure 4:**
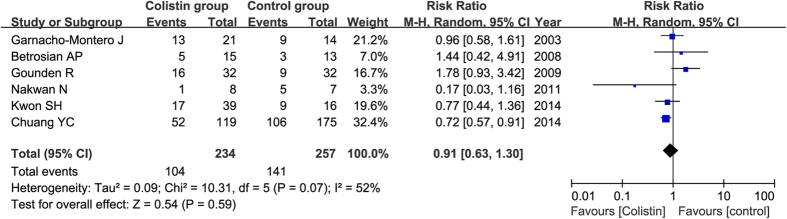
Forest plot for risk ratios in terms of mortality of colistin compared with control antibiotics.

All six studies reported the mortality, including 491 patients (234 with colistin treatment, and 257 with other antibiotics). Although the mortality rate was lower in colistin group than that in control group (43% versus 50%), no significant difference was found between these two groups (RR = 0.91, 95% CI = 0.63–1.30, P = 0.59) as shown in Fig. 4.

### Length of stay

All the studies reported the length of stay containing 491 patients. We can’t conduct a statistical analysis due to different means of expression (some articles reported the mean days of length of stay, while others reported the range of days). All of them demonstrated that there was no statistically significant difference in the median length of hospital stay between two groups (P > 0.05).

### Safety analysis

**Figure 5:**
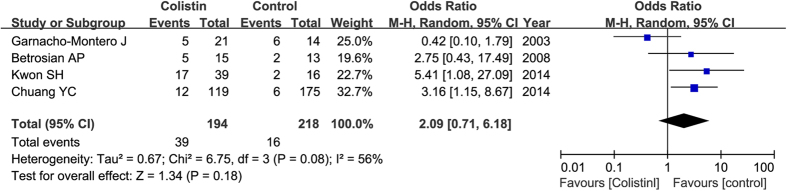
Risk of nephrotoxicity with colistin compared with control antibiotics.

The main adverse effects of colistin treatment are nephrotoxicity and neurotoxicity. Four studies, including 412 patients, reported nephrotoxicity. Overall, it did not differ significantly between colistin group and control group (OR = 2.09, 95% CI = 0.71–6.18, P = 0.18) in a random effect model as shown in Fig. 5. No studies reported the results of neurotoxicity.

### Meta-analysis of colistin-based combination therapy

#### Clinical outcome

**Figure 6:**
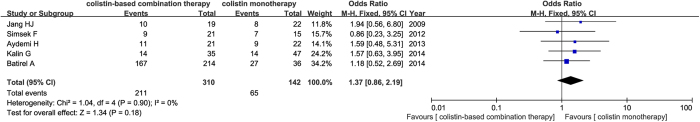
Clinical response with colistin combination therapy compared with monotherapy.

Five studies reported the clinical response, including 412 patients (194 with colistin-based combination therapy, and 218 with colistin monotherapy). The favorable clinical response was higher in patients with colistin-based combination therapy (experimental group) than that with colistin monotherapy (control group) (68.1% versus 45.8%). As shown in Fig. 6, the overall clinical response did not differ significantly between these two groups (OR = 1.37, 95% CI = 0.86–2.19, P = 0.18). No significantly heterogeneity was found between studies (I^2^ = 0%, P = 0.75).

### Microbiological response

**Figure 7:**
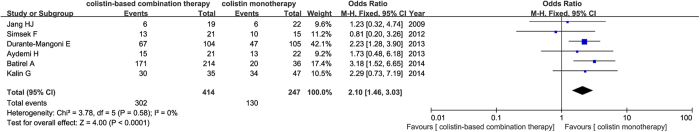
Forest plot for microbiological response between colistin combination and monotherapy groups.

All the six studies reported the microbiological response of colistin-based combination therapy (414 patients) vs. colistin monotherapy (247 patients). Our result showed a significantly association (OR = 2.14, 95% CI = 1.48–3.07, P < 0.0001) in a fixed-effects model as shown in Fig. 7.

### Mortality

**Figure 8:**
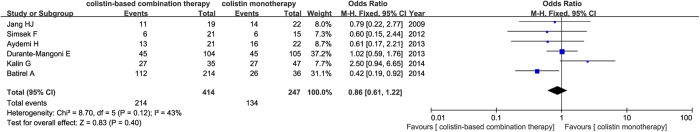
Risk ratios of mortality between colistin combination and alone groups.

All six studies reported mortality. As shown in Fig. 8, no significant difference was noted when colistin-based combination therapy was compared with colistin monotherapy with respect to hospital mortality (RR = 0.93, 95% CI = 0.74–1.17, P = 0.54) in a random-effect model.

### Length of ICU stay

Three studies reported the length of ICU stay. The mean length of hospital stay was 5.05 days (95% CI,-4.35 to 14.45 days, P = 0.29), and it did not differ significantly between the colistin-based combination therapy group and colistin monotherapy group.

### Safety analysis

Nephrotoxicity did not differ significantly between colistin-based combination therapy group and colistin monotherapy group (OR = 1.13, 95% CI = 0.74–1.73, P = 0.57).

### Sensitivity analyses and publication bias

A single study included in the meta-analysis was deleted each time to reflect the influence of the individual data set to the pooled ORs, and the corresponding pooled ORs were not materially changed. This procedure confirmed the stability of our overall result.

**Figure 9:**
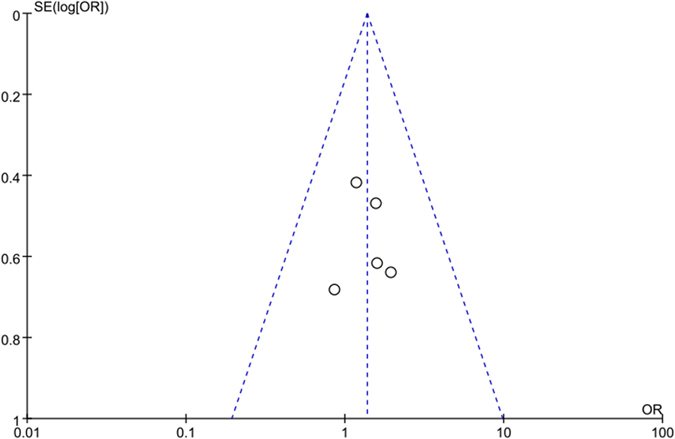
Funnel plot analysis on the detection of publication bias in the meta-analysis.

The funnel plot was conducted to assess the publication bias of the literature. The shape of funnel plots did not reveal any evidence of funnel plot asymmetry as shown in Fig. 9.

## Discussion

*A. baumannii* has emerged as a major cause of nosocomial infections. It has been identified by the Infectious Diseases Society of America as one of the six particularly problematic pathogens^24^. Due to the shortfall of effective antibiotic development for multidrug resistant *A. baumannii*, colistin is re-investigated in treating the widespread drug-resistant bacteria and has found renewed interest^25^.

Colistin has a narrow spectrum of use and is primarily used for *A. baumannii* infections. It is a multicomponent polypeptide antibiotic, composed mainly of colistin A and colistin B. Its use was limited by its renal toxicity, and it was replaced in the 1970s by antibiotics considered to be less toxic^26^. Moreover, in 2006 Li *et al.* first described colistin heteroresistance of *A. baumannii*, which was defined as the emergence of resistance to colistin by a subpopulation from an otherwise susceptible (MIC ≤ 2 mg/L) population^27^. More recently, colistin has increasingly been used as salvage therapy in combination with one or more antibacterial drugs for the treatment of severe infections in critically ill patients^7^,^28^.

Our study found that clinical response did not change significantly between colistin-based combination therapy against monotherapy AND colistin monotherapy against other antibiotic (P > 0.05). This insignificance was also found in other comparisons such as clinical outcome, mortality, length of stay, or toxicity. However, a significant difference was found between both these two comparison groups in terms of microbiological response. Our study is consistence with a previous meta-analysis conducted by Liu *et al.* which demonstrated that microbiological response rates favored the colistin group, while these differences were not significant in hospital mortality, lengths of hospital stay or nephrotoxicity in both colistin-based combination regimen vs. colistin alone AND monotherapy regimen vs. other single antibiotic groups^29^. These results suggest that colistin may be as safe and as efficacious as standard antibiotics for the treatment of *A. baumannii* infection.

In clinical practice, in order to improve antibacterial activity, colistin is frequently used as combination or single therapy, despite the consequent increase in toxicity. The comparative effectiveness of colistin monotherapy and colistin combination therapy was evaluated^30^. Most of the microbiological studies examined colistin monotherapy vs. combinations for *A. baumannii* infections. However, data from the relevant human studies suggest non-inferiority of colistin monotherapy as compared with combination therapy^31^.

Some studies have investigated the clinical effectiveness of combination therapy and have assessed the issues of the increased toxicity related to combination treatment regimens. Bassetti *et al.* found that colistin and rifampicin may be an effective and safe combination therapy for severe infections due to MDR-AB^32^. Although rifampin would be used more for synergy, research has shown that the 30-day mortality was not reduced by addition of rifampicin to colistin. Moreover, the combination of colistin with rifampicin may improve clinical and microbiological outcomes of VAP patients infected with *A. baumannii*. Cai *et al.* identified that colistin/rifampicin and colistin/carbapenem were the most studied combinations which showed promising results *in vitro*, *in vivo* and in the clinic^33^. Phee *et al.* firstly found colistin/ fusidic acid was a novel regimen for the treatment of MDR-AB, the combination was effective at low concentrations, which should be therapeutically achievable whilst limiting toxicity. Lee *et al.* showed that emergence of colistin-resistant subpopulations was completely suppressed in the colistin-susceptible isolate with all combinations at both inocula and provided important information for optimizing colistin-rifampin combinations against colistin-susceptible and -resistant MDR-AB^34^. Mutlu Yilmaz *et al.* identified that in the treatment of infections with a high mortality rate such as pneumonia caused by XDR-AB, combining tigecycline with colistin during the first 48 h and continuing treatment with one of these agents seems a rational approach^35^. However, Garnacho-Montero *et al.* demonstrated that clinical outcomes did not differ in patients treated with colistin plus vancomycin from those receiving colistin without vancomycin, and this combination significantly increases the risk of renal failure^36^.

Other studies have evaluated the effect of colistin monotherapy in severe infections caused by MDR Gram-negative pathogens. Khawcharoenporn *et al.* found that administration of primary or adjunctive intrathecal or intraventricular colistin therapy was effective for MDR-AB central nervous system infection^37^. Kang *et al.* showed that aerosolized colistin may be used as monotherapy for VAP due to *A. baumannii* infection in pre-term infants^38^. However, a major concern regarding colistin monotherapy is the potential problem of heteroresistance among Gram-negative bacterial populations exposed to colistin alone^39^.

Several limitations are presented in our meta-analysis. Firstly, the patient population enrolled and randomized was a very heterogeneous population. The population was inherently complex for the baseline conditions were diversity. Secondly, the patients were not stratified by the additional antibiotics that are known to be effective for *A. baumannii*, or antibiotics in the control group might have different effect. Thirdly, colistin drug monitoring was not available for drug-drug interactions with these medications may have affected the primary mortality. Lastly, some of the outcomes are presented in different ways, so we can’t make a statistical analysis. Furthermore, the number of included studies for a certain comparison was small.

In conclusion, our results showed that colistin could be a safe and effective alternative therapy for drug resistant *A. baumannii* infection. Although no differences were found between colistin combination therapy and colistin monotherapy AND between colistin monotherapy and other antibiotics, additional studies are needed to evaluate the effective of colistin in MDR-AB infection.

## Materials and Methods

### Literature search

We conducted a comprehensive literature search using the electronic database of PubMed, Medline and Embase for relevant articles published between January 2000 and March 2014. We retrieved the relevant articles using the following terms: “colistin or polymyxin E”, “combination therapy”, “monotherapy”, “*Acinetobacter baumannii* or *A. baumannii* infection” and “ventilator associated pneumonia or VAP” as well as their combinations. References of retrieved articles were searched with no language restrictions. The search was focused on studies that had been conducted in humans.

### Study selection

The inclusion criteria were as follows: 1) the paper should be randomized controlled trials or cohort studies; 2) evaluating the safety and efficacy of colistin monotherapy against other antimicrobial agents, or colistin combination therapy against colistin monotherapy for the treatment of *A. baumannii* infection; 3) multi-drug resistant *A. baumannii* was defined as *A. baumannii*, which showed non-susceptibility to ≥1 agent in ≥3 antimicrobial categories^40^; and 4) the primary outcome were clinical response and microbiological response (clinical response was defined as complete or partial remission of the signs and symptoms of infection by the end of therapy; microbiological response was defined as negative of culture result at the end of therapy); the secondary outcome were mortality and colistin toxicity/adverse effect (neurotoxicity, nephrotoxicity) (Neurotoxicity was defined as any of the following: seizures, encephalopathy, neuromuscular blockade and apnea; Nephrotoxicity was defined as initiation or as a decline in renal function that prompted renal replacement therapy).

### Data extraction

Two investigators independently assessed the quality of the included studies according to the descriptions provided by the authors of the included trials. Any disagreement was subsequently resolved by discussion with a third author. The following information was extracted from each article: first author, year of publication, country, ethnicity, number of patients, type of colistin administered, co-administration of other antibiotics, clinical response, mortality, length of therapy and number of patients with nephrotoxicity.

### Statistical analysis

The overall effect was measured by odds ratios (ORs), risk ratios (RRs), and mean difference (MD) with their 95% confidence interval (CI), which were calculated according to the method of Woolf^41^. The significance of the pooled ratios was determined by the Z test, and a P value less than 0.05 was considered statistically significant. The I^2^ test was used to assess the proportion of statistical heterogeneity and the Q-statistic test was used to define the degree of heterogeneity. A P-value less than 0.10 for the Q-test and I^2^ more than 50% was considered significant among the studies. Data were combined using both a fixed-effects model and a random-effects model^42^,^43^. The fixed-effects model is used when the effects are assumed to be homogenous, while the random-effects model is used when they are heterogenous. The evidence of publication bias was assessed by visual funnel plot inspection.

To assess whether our results were substantially influenced by the presence of any individual study, we conducted a sensitivity analysis by systematically removing each study and recalculating the significance of the result. Statistical analyses were conducted in Review Manager (version 5.2, The Cochrane Collaboration). All the tests were two-sided.

## Additional Information

**How to cite this article**: Chen, Z. *et al.* Meta-analysis of colistin for the treatment of *Acinetobacter baumannii* infection. *Sci. Rep.* **5**, 17091; doi: 10.1038/srep17091 (2015).

## Figures and Tables

**Table 1:** Characteristics of the included studies.

First author	Year	Country	Type of study	Organisms isolated group	Experimental			Control group		
					Antibiotics Sample size		Age	Antibiotics		
								Age Sample size		
Colistin monotherapy										
Garnacho-Montero J	2003	Spain	Prospective cohort	AB	Colistin	59.3 ± 13.1	21	Imipenem-cilastain	64.5 ± 11	14
Betrosian AP	2008	Greece	Prospective cohort	MDRAB	Colistin	67 ± 9	15	Ampicillin/Sulbactam	72 ± 5	13
Gounden R	2009	South Africa	Retrospective cohort	MDRAB	Colistin	43.5 ± 15.6	32	Tigecycline	45.6 ± 18.2	32
Nakwan N	2011	Thailand	Retrospective cohort	EDRAB	Colistin	38 (28–41)	8	Other antibiotics	29 (28–34)	7
Chuang YC	2014	China Taiwan	Retrospective cohort	MDRAB	Colistin-based	63.7 ± 19.5	119	Tigecycline-based	63.8 ± 17.9	175
Kwon SH	2014	Korea	Retrospective cohort	EDRAB	Colistin	59.0 ± 19.2	39	Tigecycline	60.1 ± 12.3	16
Colistin combination therapy										
J ang HJ	2009	Korea	Retrospective cohort	MDRAB	Colistin/synergistic antibiotics	57.0 ± 16.5	19	Colistin	62.5 ± 17.5	22
Simsek F	2012	Turkey	Retrospective cohort	AB	Colistin-based	51.7 ± 18.8	21	Colistin	51.7 ± 18.8	15
Aydemi H	2013	Turkey	RCT	CRAB	Colistin/ Rifampicin	58 ± 23	21	Colistin	63 ± 17	22
Durante-Mangoni E	2013	Italy	RCT	EDRAB	Colistin/ Rifampicin	62 ± 15.1	104	Colistin	61 ± 15.7	105
Batirel A	2014	Turkey	Retrospective cohort	EDRAB	Colistin/other antibiotics	59.1 ± 19.6	214	Colistin	58.3 ± 20.5	36
Kalin G	2014	Turkey	Retrospective cohort	MDRAB	Colistin/Sulbactam	63 (20–89)	37	Colistin	51 (19–96)	52

RCT, randomized controlled trial; AB, Acinetobater baumannii; MDRAB, multi-drug resistant Acinetobater baumannii; EDRAB, extensively drug resistant Acinetobater baumannii; CRAB, carbapenem-resistant Acinetobater baumanni.
